# Supplementary material for: Architecture of the sperm whale forehead facilitates ramming combat
Source: PeerJ. 2016 Apr 5;4:e1895. doi: 10.7717/peerj.1895 (PMC4824896; doi:10.7717/peerj.1895)
Supplement: Supplemental Information 2 [file peerj-04-1895-s002.pdf]

BASE CASE (Model A) – Reaction forces, horizontal

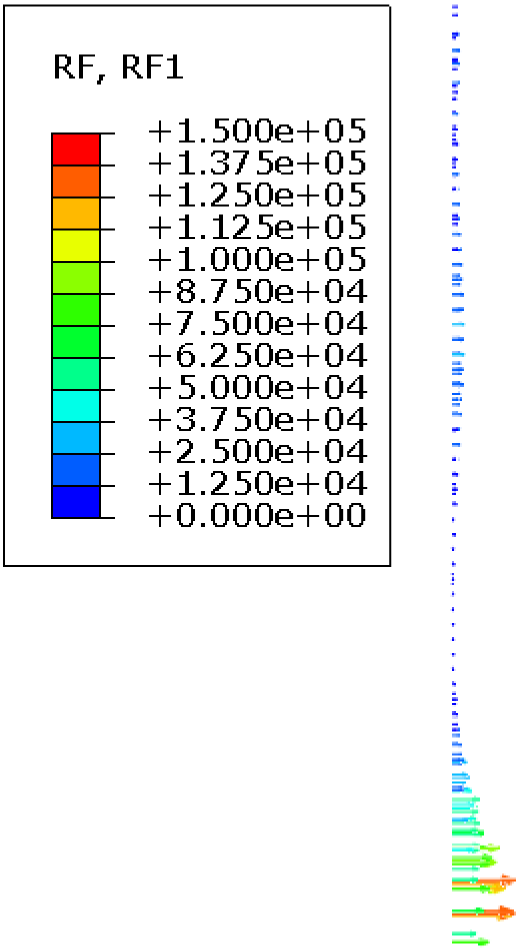

TOTAL = 9.56016E+06

HALF PARTITIONS (Model B) – Reaction forces, horizontal

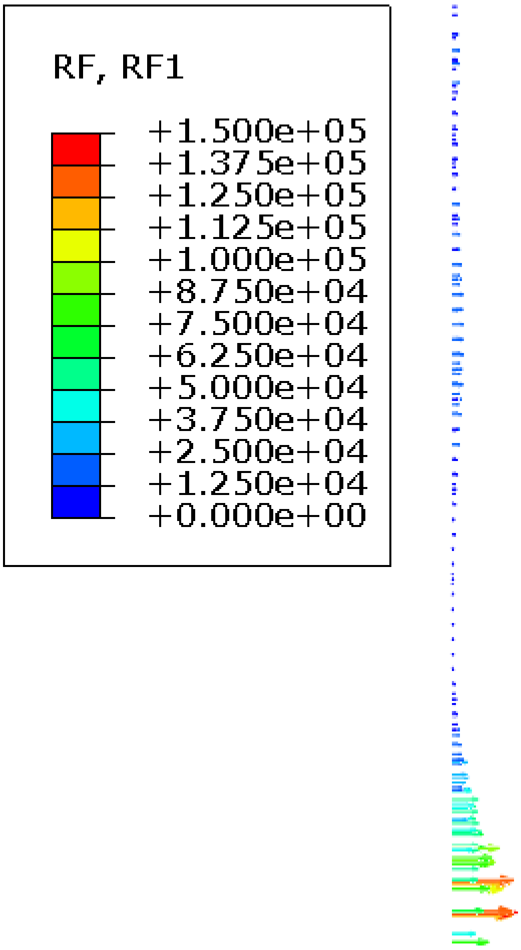

TOTAL = 9.16811E+06

NO Parttions (Model C) – Reaction forces, horizontal

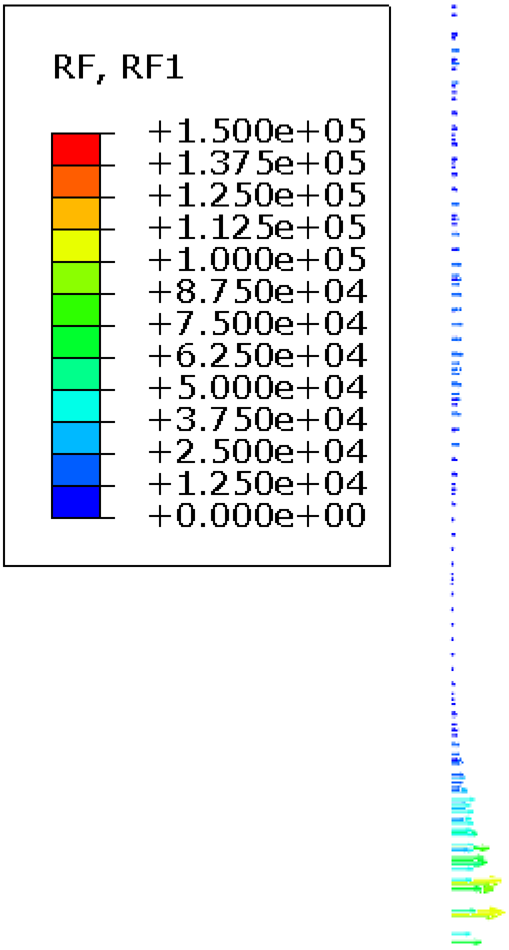

TOTAL = 7.80574E+06
