# Supplementary figures and images for: Architecture of the sperm whale forehead facilitates ramming combat
Source: PeerJ. 2016 Apr 5;4:e1895. doi: 10.7717/peerj.1895 (PMC4824896; doi:10.7717/peerj.1895)

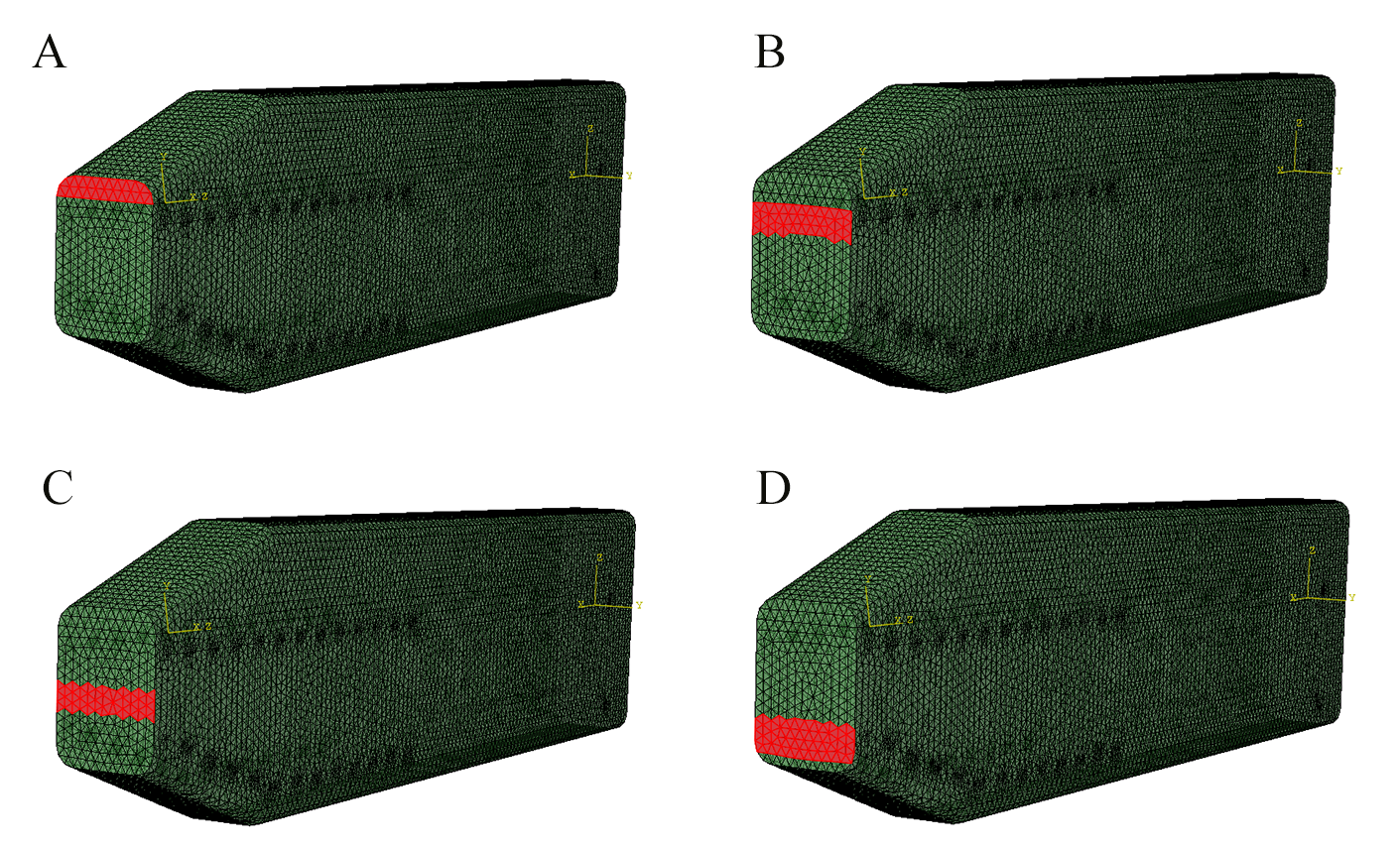

Supplement: Figure S1 — (A) Impact force on the spermaceti organ; (B) Impact force on the superior aspect of the spermaceti junk; (C) Impact force on the mid spermaceti junk; (D) Impact force on the inferior aspect of the spermaceti junk. [file peerj-04-1895-s004.png]

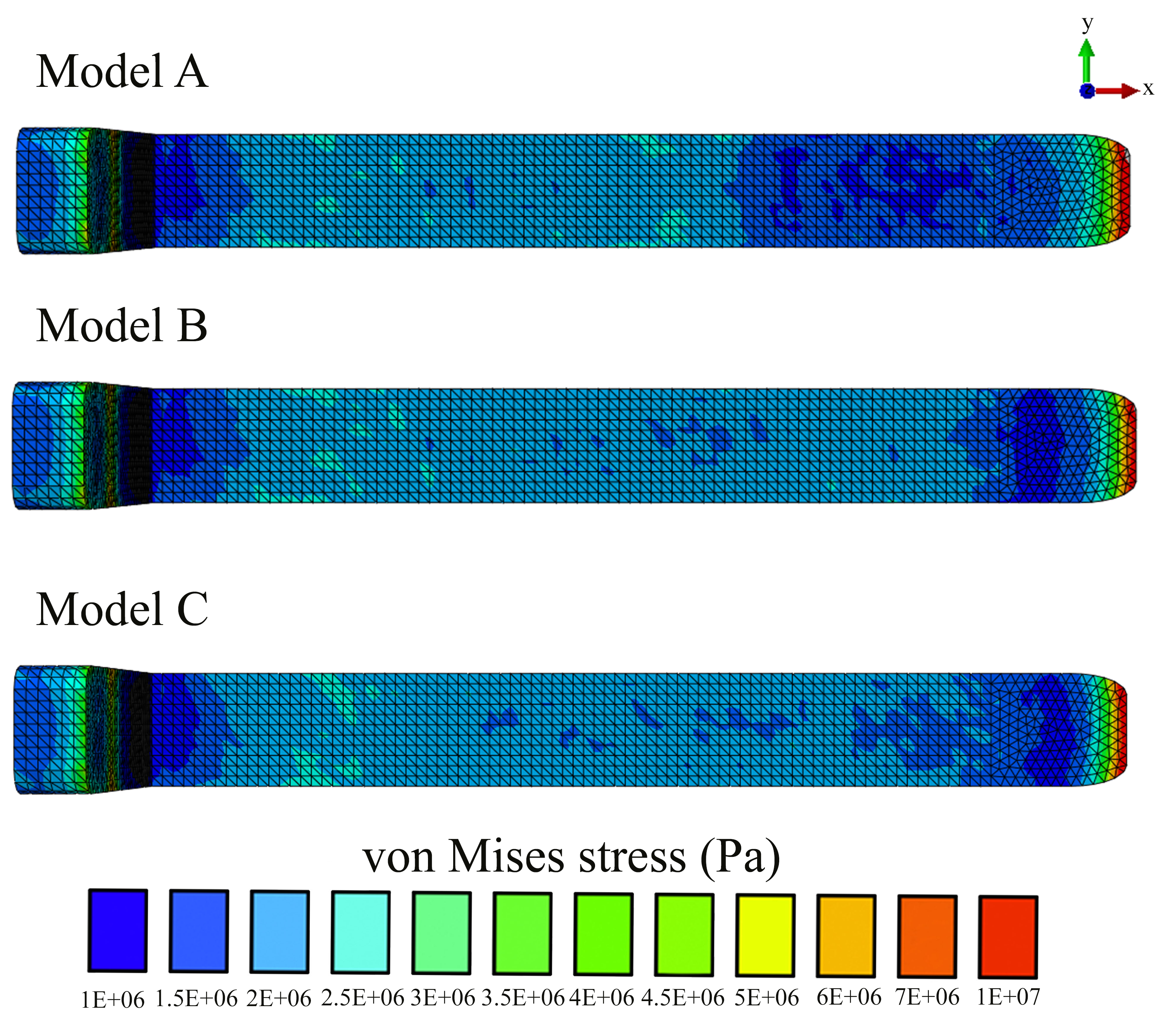

Supplement: Figure S2 [file peerj-04-1895-s005.png]

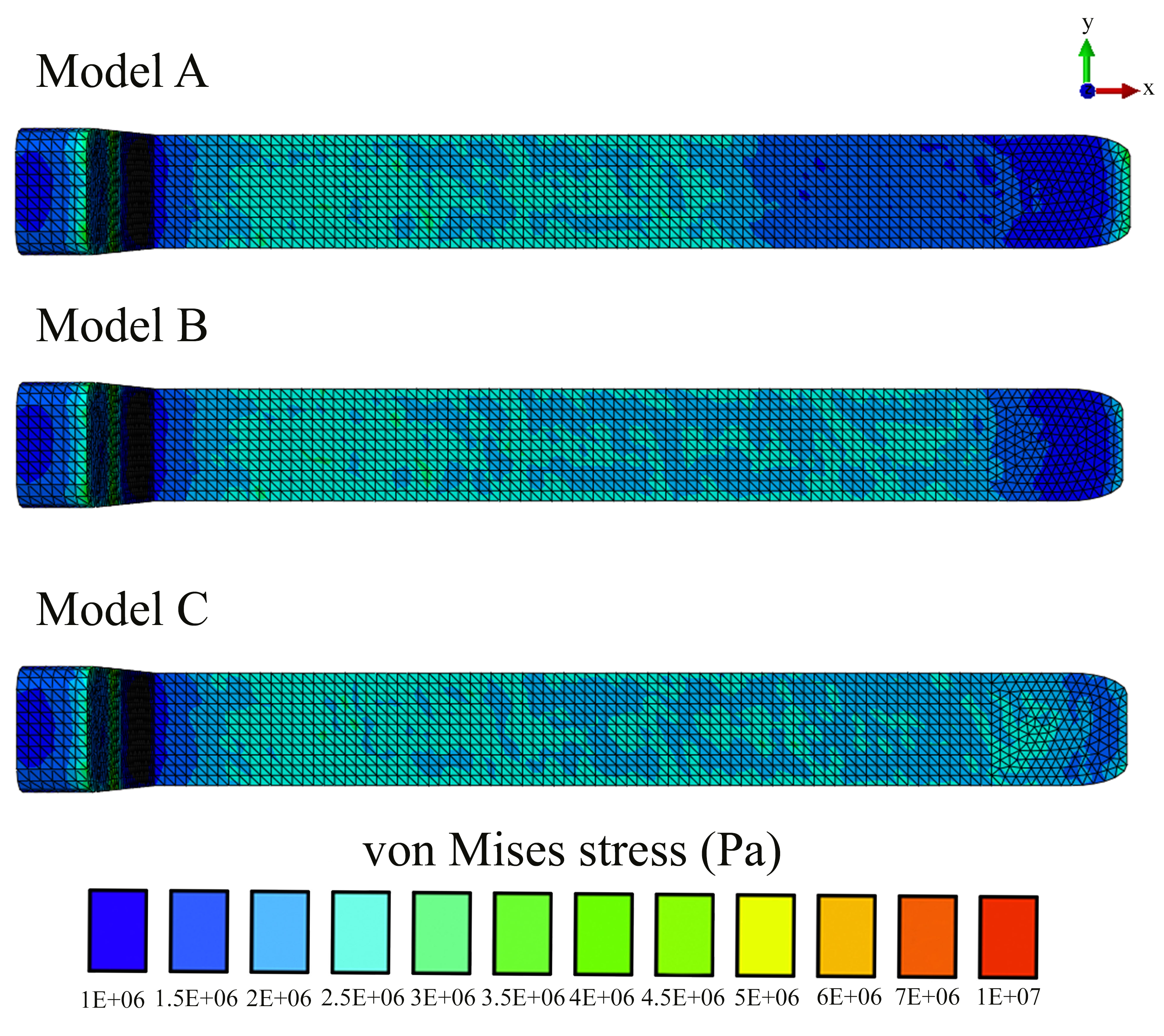

Supplement: Figure S3 [file peerj-04-1895-s006.png]

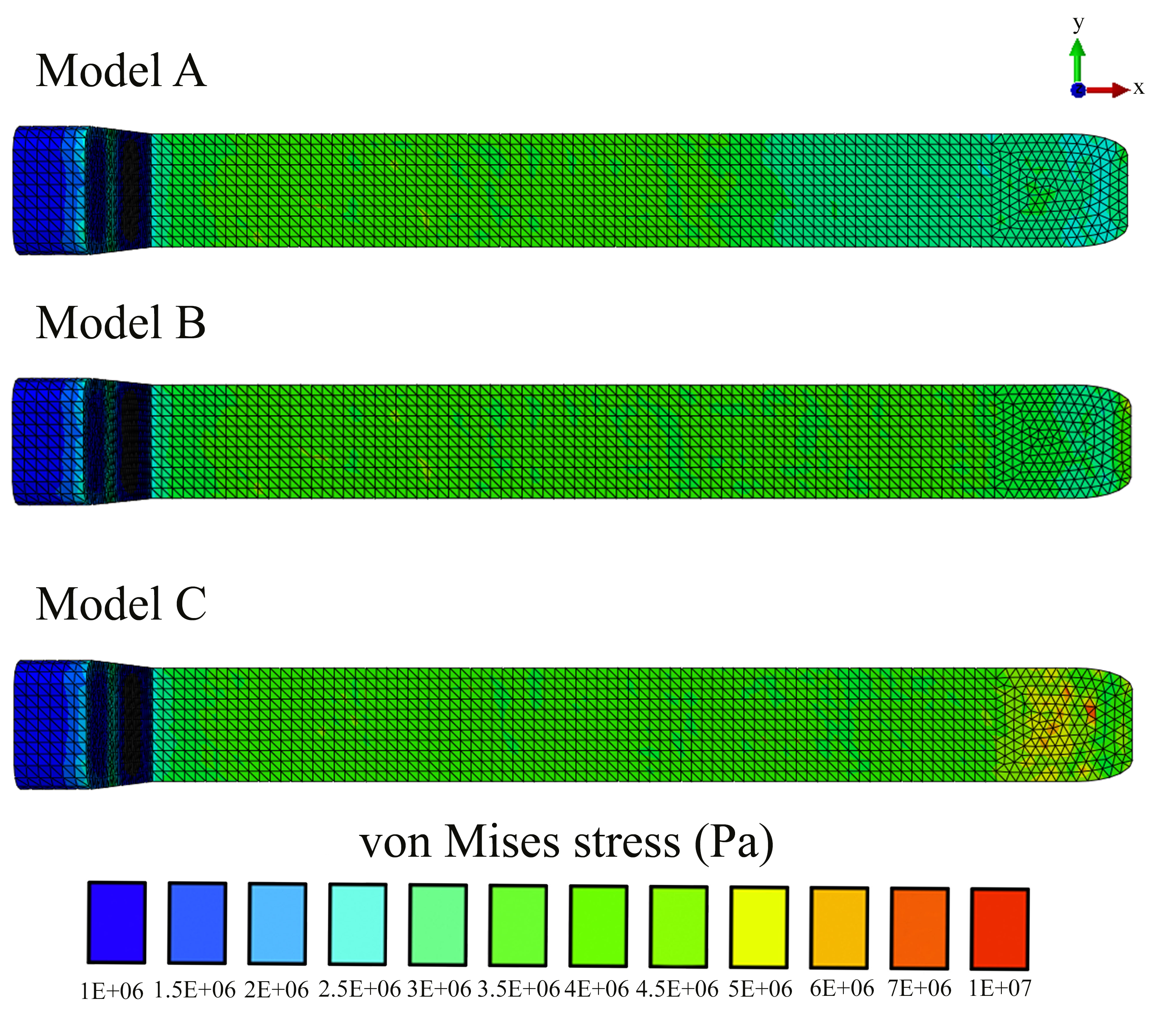

Supplement: Figure S4 [file peerj-04-1895-s007.png]

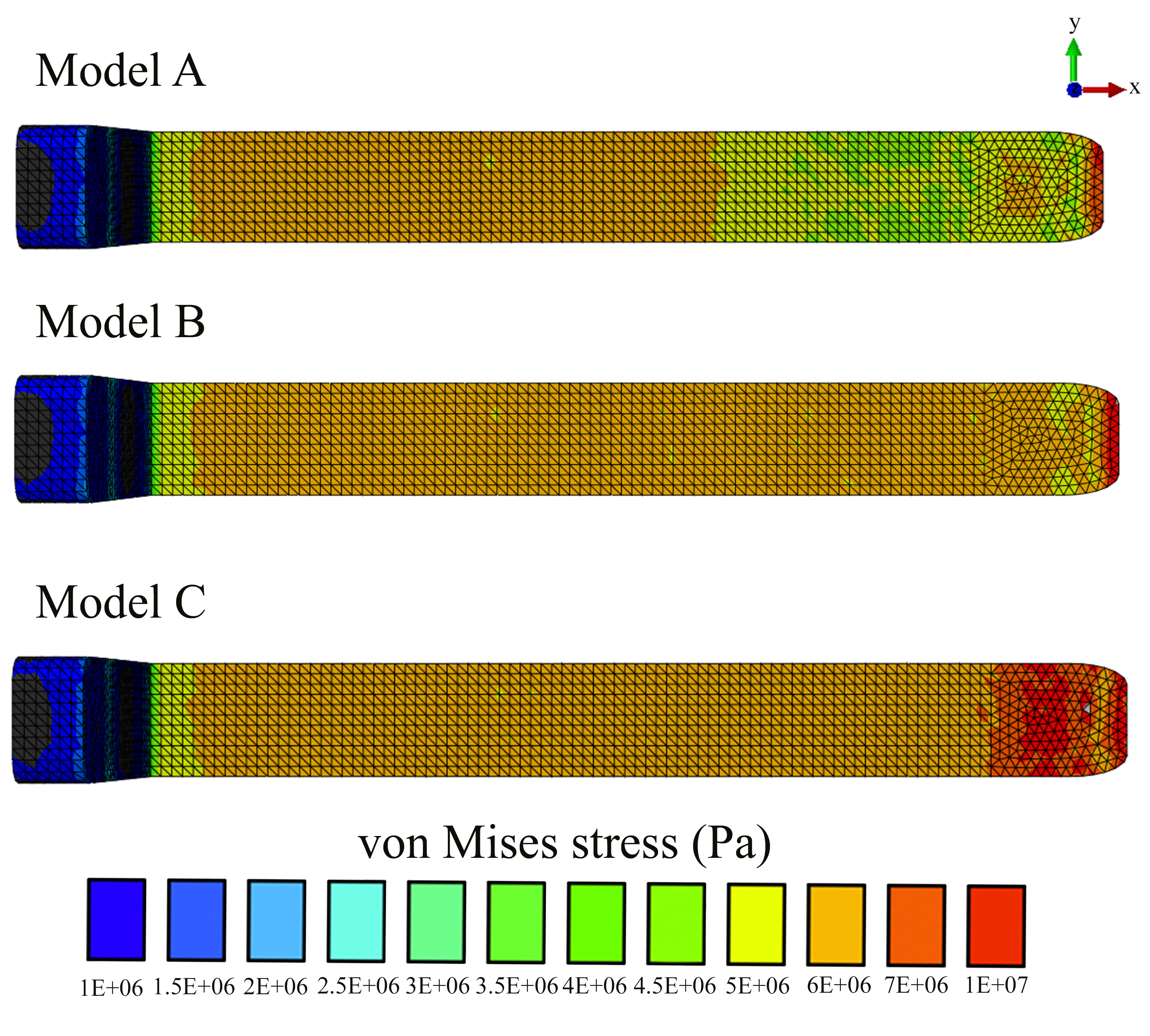

Supplement: Figure S5 [file peerj-04-1895-s008.png]

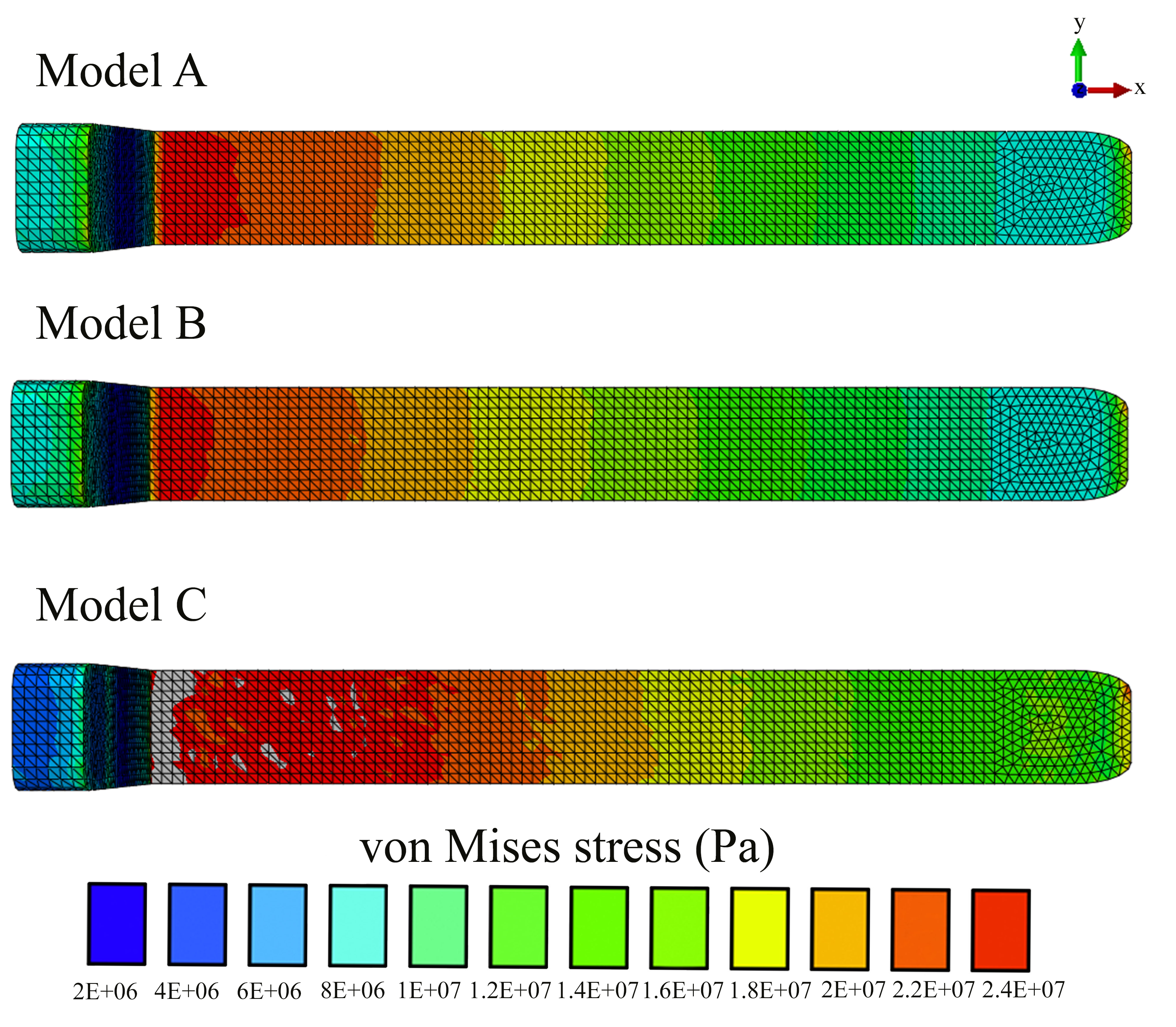

Supplement: Figure S6 [file peerj-04-1895-s009.png]

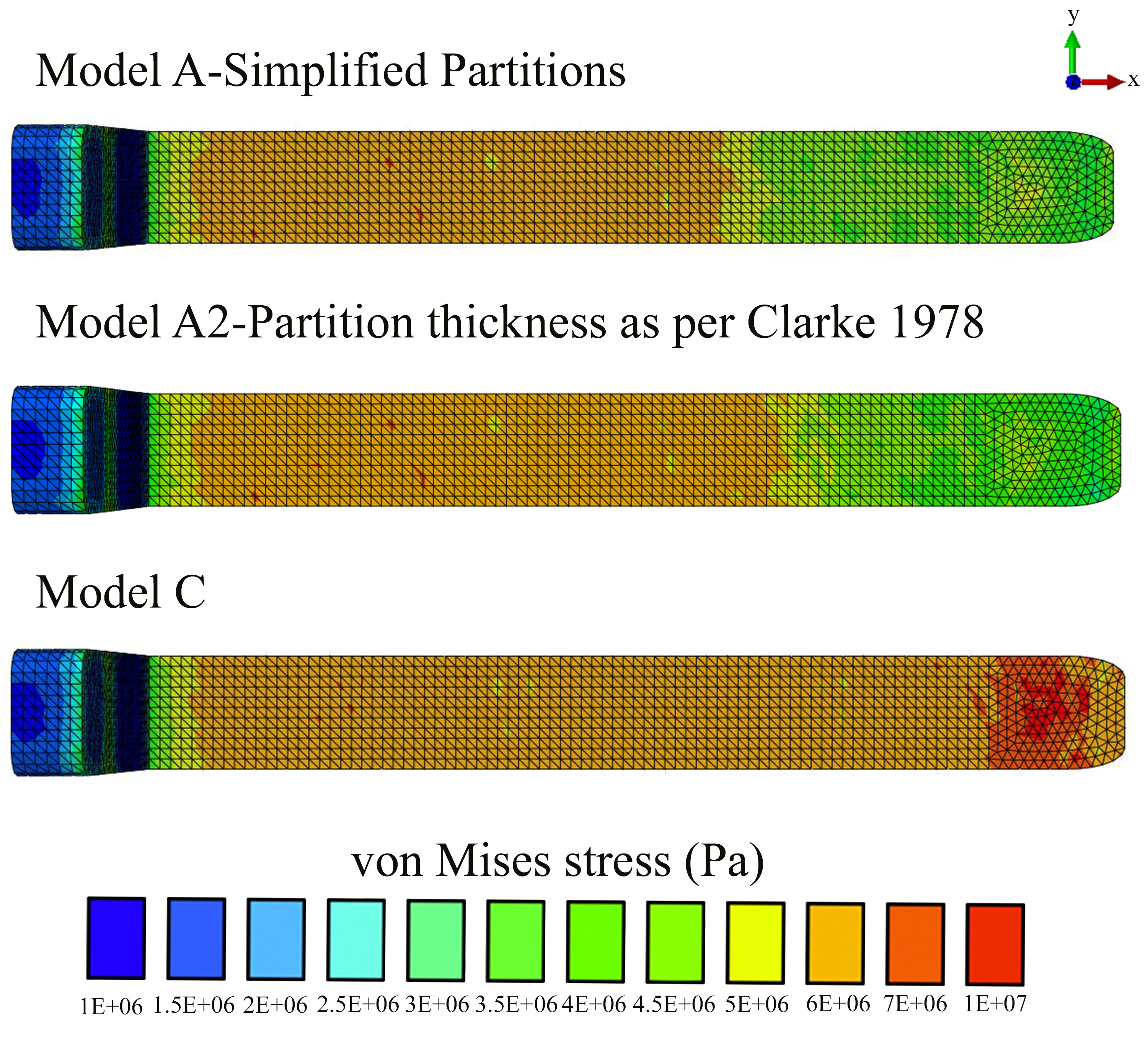

Supplement: Figure S7 [file peerj-04-1895-s010.png]

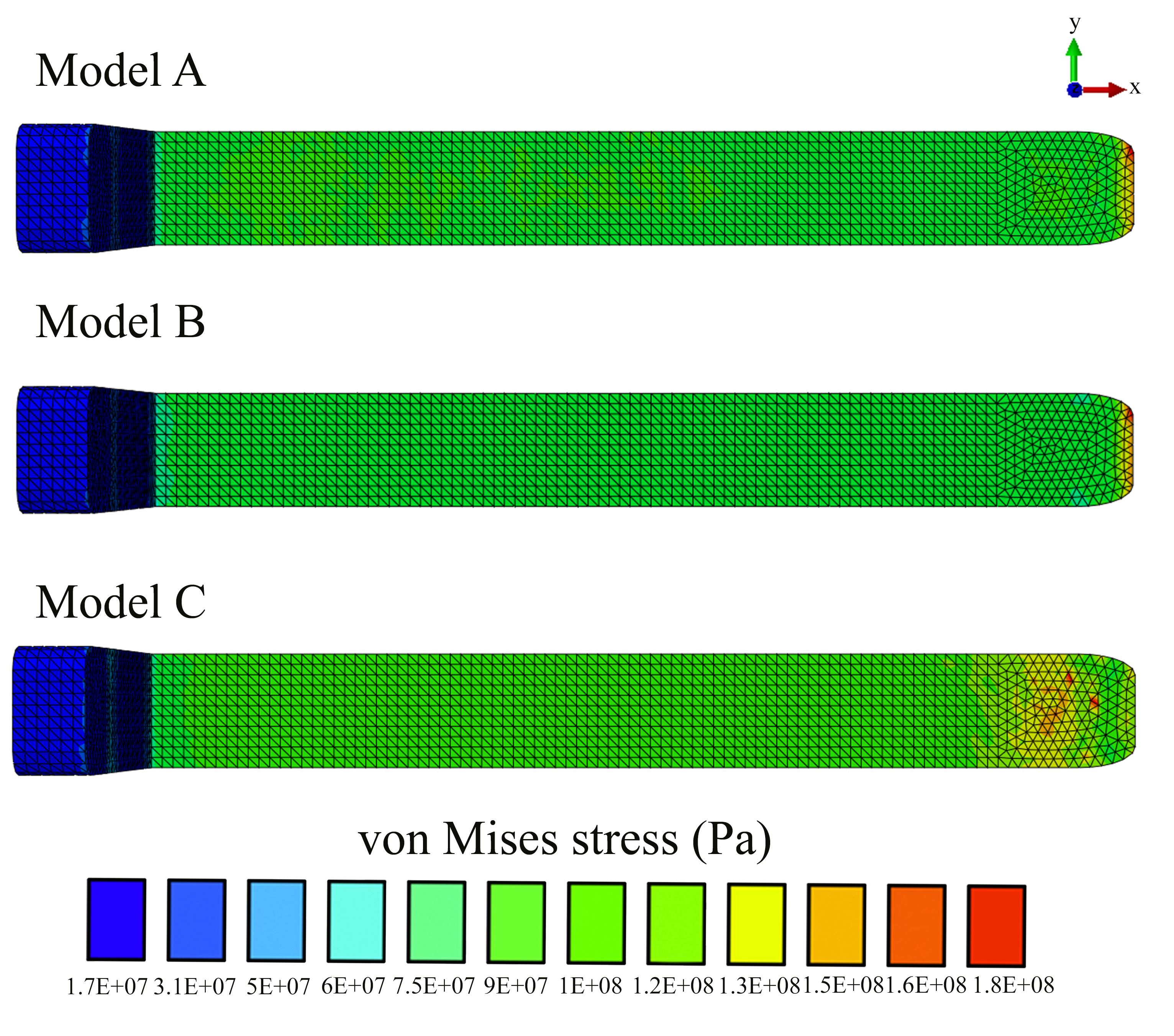

Supplement: Figure S8 [file peerj-04-1895-s011.png]
