# Supplementary material for: Architecture of the sperm whale forehead facilitates ramming combat
Source: PeerJ. 2016 Apr 5;4:e1895. doi: 10.7717/peerj.1895 (PMC4824896; doi:10.7717/peerj.1895)
Supplement: Table S1 [file peerj-04-1895-s012.docx]

**Table S1.** Magnitude of the force assigned to all FEMs to evaluate the sensitivity of the skull stresses on variations of the topological application of the impact force (Figure S1).

| **FORCE SECTION** | **AREA (sq.m)** | **TOTAL FORCE (N)** | **PRESSURE APPLIED (Pa)** |
| --- | --- | --- | --- |
| SPERMACETI ORGAN (SO) | 0.0647 | 764,000 | 1.18E+07 |
| SPERMACETI JUNK (SJ) TOP | 0.11 | 764,000 | 6.95E+06 |
| SPERMACETI JUNK (SJ) MIDDLE | 0.1003 | 764,000 | 7.62E+06 |
| SPERMACETI JUNK (SJ) BOTTOM | 0.1359 | 764,000 | 5.62E+06 |

**Table S2.** Connective tissue partitions thickness (m) approximation reported by Clarke (1978) and simplified for the scope of this project.

| Partitions | Height (m) | | Width (m) | | Case Width (m) | |
| --- | --- | --- | --- | --- | --- | --- |
| Posterior-Anterior | Clarke | Simplified | Clarke | Simplified | Clarke | Simplified |
| 1 | 0.27 | 0.41 | 0.04 | 0.15 | 0.04 | 0.10 |
| 2 | 0.44 | 0.48 | 0.05 | 0.15 | 0.03 | 0.05 |
| 3 | 0.64 | 0.56 | 0.08 | 0.15 | 0.05 | 0.05 |
| 4 | 0.72 | 0.63 | 0.16 | 0.15 | 0.05 | 0.05 |
| 5 | 0.72 | 0.71 | 0.15 | 0.15 | 0.06 | 0.05 |
| 6 | 0.71 | 0.73 | 0.17 | 0.15 | 0.06 | 0.05 |
| 7 | 0.70 | 0.73 | 0.17 | 0.15 | 0.08 | 0.05 |
| 8 | 0.67 | 0.73 | 0.17 | 0.15 | 0.08 | 0.05 |
| 9 | 0.64 | 0.73 | 0.17 | 0.15 | 0.08 | 0.05 |
| 10 | 0.62 | 0.73 | 0.17 | 0.15 | 0.08 | 0.05 |
| 11 | 0.58 | 0.73 | 0.10 | 0.15 | 0.04 | 0.05 |
| 12 | 0.55 | 0.73 | 0.10 | 0.15 | 0.03 | 0.05 |
| 13 | 0.52 | 0.73 | 0.10 | 0.15 | 0.03 | 0.05 |
