# Supplementary material for: Architecture of the sperm whale forehead facilitates ramming combat
Source: PeerJ. 2016 Apr 5;4:e1895. doi: 10.7717/peerj.1895 (PMC4824896; doi:10.7717/peerj.1895)
Supplement: Supplemental Information 1 — Report by Sandra Lanham on ramming sperm whales [file peerj-04-1895-s001.pdf]

Environmental Flying Services
MEXICO-UNITED STATES

8 December 2015

To: Dr. Olga Panagiotopoulou, School of Biomedical Sciences, The University of Queensland.

Dear Dr. Panagiotopoulou,

I am writing to share a report written for Environmental Flying Services on January 31st, 1997, the day after I witnessed a sperm whale ramming incident near the Midriff Islands in the Gulf of California, Mexico. I also give my permission to publish this report in support of the manuscript entitled “Architecture of the sperm whale forehead facilitates ramming combat”.

Although now retired, on the day of the sighting I was a wildlife pilot and the director of Environmental Flying Services, a non-profit organization created to help wildlife researchers. At the time of this observation, I had logged about 3500 hours of research flights, with 70 to 80 percent of those hours spent over the Gulf of California working with biologists studying fisheries, marine mammals, and sea turtles, among other species. In 2001, based on this work, I was named a MacArthur Fellow.

Original Report:

DATE: January 30, 1997.
LOCATION: Midriff Islands, Gulf of California, Mexico 28˚ 34.350 112˚ 36.201
CONDITIONS: Beaufort 0 to 1, 5-10 MPH southwest winds, visibility 75 miles.
REPORTED BY: Sandy Lanham, Environmental Flying Services

I was flying over the Gulf of California at 7500 feet in a Cessna 182, eastbound to Hermosillo, Sonora returning from the Pacific coast of Baja California.

A few miles southwest of the southern end of San Esteban Island, I saw two whales approaching one another on the surface. When I first saw them, they were an estimated four miles apart converging head to head on an approximate east-west line. The whale nearest me was perhaps 30% larger than the other. He was so big that, at first, I thought he was a blue whale. Using binoculars (7x50), I identified both whales as sperm whales. They were light gray in color but sometimes appeared blue underwater. A number of sperm whales, some with calves, were just a few miles to the south between San Esteban Island and San Pedro Martir Island, roughly 50 of them.

5900 South Camino de la Tierra
Tucson, Arizona 85746
520.578.5610
www.eflying.org

I descended to 2000 feet, an altitude chosen in order to see better while not disturbing the whales, and flew to each converging whale to mark its position with a GPS waypoint. Based on the size and shape of the head, I identified each whale as an adult male. With these position coordinates, and the times recorded for each by the GPS, the distances between the whales and their speeds could later be calculated. Then I circled, waiting to see what would happen. Fin whales were nearby, as were feeding dolphins (probably common dolphins).

The converging whales swam to one another without obvious speed changes or deviations in course, making occasional shallow dives. About 20 feet from one other, the smaller one shallow dove and a split second later, the big one did also. Leveling under water, they rammed head to head. There was surprisingly little splash on the surface. One slid to the side of the other so that their heads and upper third of their bodies overlapped. They rolled slightly, turning their bellies toward one another. It appeared that each had his mouth opened wide. It was my impression that they were trying to lock jaws or to bite one another's head. At the exact moment their heads touched, I took another waypoint.

After about five seconds, the whales dove out of sight without fluking. They left a single footprint in an unbroken sea surface. I continued circling, waiting. After about a minute, both rose to the surface and swam to the southeast in the direction of the other sperm whales. The smaller one trailed the big one by a whale's length.

On my marine radio, I called Tad Pfister of the Prescott College Research Station in nearby Kino, picked him up at the local airstrip, and we returned by air to the location of the ramming. Tad works with whales in the Midriff Islands, including sperm whales. After looking for perhaps 30 minutes, a bull sperm whale surfaced under us and spouted. We recorded him one and a half hours after the ramming. He was less than one nautical mile (.98nm) from its site.

He was one of the biggest sperm whales either of us had seen. He looked bigger than a nearby fin whale. We'll never know if he was one of the bulls involved in the ramming but we operated as though he was. Tad took photos at 500 feet as indicated on the airplane's altimeter (not an accurate attitude without a local pressure setting). He asked me to climb slightly because he could not fit the whale in the frame of his 200 mm lens. This combination of altitude and lens works well for blue whales.

Once home, I calculated the times and distances. Based on the GPS coordinates and the times each waypoint was recorded, the whales were approximately four miles apart when I first saw them and it took them 11 to 12 minutes to reach one another and ram. Assuming they both were traveling at the same rate, their speed therefore was roughly 10 to 11 MPH. Whale researchers tell me this is top speed for sperm whales.

Tad went out the next day in his panga (skiff) to investigate. He found approximately 60 sperm whales in the same general area, including males, females, and calves. Their general behavior, he told me, indicated possible mating activity.

Sincerely,

Ms. Sandra Lanham
